# 21063 A Review of Novel Uses of REDCap in Clinical and Translational Science

**DOI:** 10.1017/cts.2021.529

**Published:** 2021-03-30

**Authors:** Aaryn Toles, Barbara Tafuto, Doreen Lechner

**Affiliations:** 1 Rutgers Robert Wood Johnson Medical School; 2 Rutgers School of Health Professions

## Abstract

ABSTRACT IMPACT: This review will encourage further development of novel uses of REDCap for the benefit of the research community. OBJECTIVES/GOALS: REDCap is a clinical research data collection platform that is primarily used as intended. However, little is known about its more novel uses, specifically in clinical decision support in patient care and in clinical research management. Thus, the purpose of this review is to examine peer reviewed literature identifying and describing such novel uses. METHODS/STUDY POPULATION: A systematic search was conducted in both PubMed and Google Scholar using the equation ((REDCap) OR ('Research Electronic Data Capture')) AND ((Clinical Trial Management) OR (Clinical Research)).’ Articles were screened by title, then abstract, and then were reviewed in full if they met inclusion criteria. Articles were included if they had potential relevance to the topic of REDCap or if they mentioned activities related to fields of clinical and translational science including operational support in areas such as clinical research management. Articles were excluded if they focused on common clinical research activities relating to data collection software such as survey administration, database building or data collection for clinical trials, registries, and cohort studies. RESULTS/ANTICIPATED RESULTS: The initial search yielded 390 results, of which 40 underwent an abstract review; only 8 of these underwent full text review. Of these, 5 discussed uses of REDCap in the context of operational support in clinical research management; 3 were related to clinical decision support in patient care. For the 5 articles focused on operational support in clinical research management, topics include e-consenting procedures, collection and storage of protected health information (PHI), patient recruitment and tracking stakeholder engagement. The 3 articles about clinical decision support discuss REDCap tools for generating risk predictions for post-surgical clinical outcomes, generating recommendations and STI test orders, and increasing efficiency in hand-offs to enhance care of surgical oncology patients. DISCUSSION/SIGNIFICANCE OF FINDINGS: Considering that only a small percentage of peer reviewed research reports out on novel uses of REDCap, there is a need for the REDCap consortium to do further work to fulfill its mission to adopt, innovate, and suggest novel uses of REDCap, thus expanding the understanding of its functionalities and therefore its utility in the research community.

